# Case of Strangulated Ventral Hernia, with Successful Operation

**Published:** 1826-11

**Authors:** 

**Affiliations:** St. Thomas's Hospital.


### VENTRAL HERNIA.

### *Case of Strangulated Ventral Hernia, with successful Operation.* Treated by Mr. Green, at St. Thomas's Hospital.

Elizabeth Garratt, a widow, fifty-nine years of age, and of corpulent habit, was admitted September 22d, 1826. When she came in (at five p.m.) the feet were cold, and she complained of general chilliness; her tongue was very dry and foul, and her pulse small, but quick and wiry. She vomited a brownish yellow fluid, intermixed with dark portions of solid matter, which gave out a feculent smell. The vomiting was repeated at intervals of a quarter of an hour, accompanied by hiccough. Upon examination of the abdomen, a flattened diffused tumor, of a red colour, was found just above the umbilicus : it was of firm texture, painful, and tender when handled, and perhaps about the size of a goose-egg. The abdomen generally, however, was lax, and free from tenderness.

She stated that for fourteen years she had been the subject of hernia, which was originally of the size of the tip of one's thumb, but which had always been readily reducible. It seems that she had likewise been afflicted with rheumatic gout, and that she had been confined to her bed for some months past, in consequence of it.

The tumor above mentioned suddenly became protruded on the 18th, when she immediately applied to a medical man, who placed a bandage around the abdomen, but which could not be borne, from its producing great pain. From this day the vomiting continued unremittingly; there having been no evacuation from the bowels since the 15th, excepting a little feculent matter brought away after the administration of a glyster on the 21st.

Fourteen ounces of blood having been abstracted from the arm, and the patient being placed so as to relax the abdominal muscles, the taxis was gently and cautiously employed. This, however, from the firmness of the tumor, and from the impossibility of determining where the pressure ought to be directed, was unsuccessful. The nature of the case was then pointed out to the patient, and she readily consented to have an operation performed. Accordingly, at half-past seven, having been placed in a convenient position, an incision was made through the integuments, in the transverse direction of the tumor, and another perpendicular to it, but extending a little beyond. The coverings of the hernia were extremely thin, there not being any proper fascia superficialis. On opening the sac, a large mass of omentum made its appearance, adherent in many places to the sides of the sac, and completely enveloping a knuckle of intestine, measuring about three inches. The gut was of a dark lake hue, was distended with flatus, and had some lymph effused on its surface. The aperture of the sac only admitted the tip of the little finger, but, when dilated by means of an incision, the bowel was returned. A considerable portion of omentum, probably of the size of a hen's egg, was removed. Some bleeding ensued from the divided vessels of the omentum, which was restrained by the application of two ligatures. The integuments were brought in apposition, and retained by means of two sutures, and lint and adhesive straps applied. Upon her return to bed after the operation, her pulse became quite soft, but the blood previously drawn was now buffed.

Next day (23d), the bowels not having been moved, Ol. Ricini ℥ss. ex Aquæ Menth. was ordered, which procured a slight motion. It was repeated, and copious good motions ensued. She had slept a little during the night; her tongue was much less furred, and the vomiting had subsided soon after the operation. She complained of soreness and aching of the wound; in consequence of which, the abdomen was ordered to be fomented. In the evening she became very restless and feverish, and was bled by the dresser to twelve ounces; after which she slept comfortably. On the 25th, she complained of a good deal of pain in the abdomen, and tenderness near the wound, with a sense of tightness: the castor-oil was repeated, and she was bled to eight ounces.

On the 2d October, the lips of the wound were in a sloughing condition, and the omentum beneath appeared in the same state. The smell of the parts was extremely offensive: her spirits were depressed, her features pinched, and her appetite bad. She was therefore ordered porter, sago, and wine, with the Pil. Rhæi ter die. On the 4th, the sloughs had separated, but matter in some quantity lodged beneath the integuments. Her appetite improved. On the 6th, a still further amendment was observed, and the ligatures came away.

She has had an attack of gout in her hands, which has now subsided.

On the 16th, the matter was discharged in much less quantity; the wound nearly healed, and the patient going on perfectly well.

The above is not an unusual case, but is rendered interesting by the patient's recovery under circumstances that would have warranted an unfavourable prognosis. In this case, the circumstances of the patient's age, habits of life, condition of body, and previous state of health, together with the length of time during which the hernia had been strangulated, would have justified doubts of her recovery. The writer has known several instances in which, under far more favourable appearances, middle-aged women have sunk after the operation for strangulated umbilical hernia, and where nothing was detected in the examination after death, that could have accounted for the event.

